# Case Report: Metagenomic Next-Generation Sequencing Clinches the Diagnosis of Acute Q Fever and Verified by Indirect Immunofluorescence Assay

**DOI:** 10.3389/fmed.2022.846526

**Published:** 2022-05-26

**Authors:** Yide Yang, Qingmiao Shi, Qian Jin, Zhangnv Yang, Wangfang Li, Jianfeng Han, Juanjuan Mao, Beiwen Zheng

**Affiliations:** ^1^Department of Infectious Diseases, Taizhou Municipal Hospital, Taizhou, China; ^2^Jinan Microecological Biomedicine Shandong Laboratory, Jinan, China; ^3^State Key Laboratory for Diagnosis and Treatment of Infectious Diseases, Collaborative Innovation Center for Diagnosis and Treatment of Infectious Diseases, The First Affiliated Hospital, College of Medicine, Zhejiang University, Hangzhou, China; ^4^Department of Microbiology, Zhejiang Provincial Center for Disease Control and Prevention, Hangzhou, China; ^5^Realbio Genomics Institute, Shanghai, China; ^6^Sansure Biotech Inc. Medical Affairs Department, National Joint Local Engineering Research Center for Genetic Diagnosis of Infection Diseases and Tumours, Beijing, China

**Keywords:** Q fever, *Coxiella burnetii*, metagenomic next-generation sequencing, indirect immunofluorescence assay, case report

## Abstract

Q fever is a zoonotic infectious disease caused by *Coxiella burnetii*. The clinical symptoms of acute Q fever are usually atypical, and routine serological tests of *C. burnetii* are not readily available, making the diagnosis of Q fever a challenge. In this case, we report a male patient who had repeated fevers and was administered empirical anti-infective treatment, but the effect was poor. After conducting relevant laboratory and imagological examinations, the etiology has not yet been confirmed. Subsequently, metagenomic next-generation sequencing (mNGS) identified the sequence reads of *C. burnetii* from the patient's peripheral blood within 48 h, and then the diagnosis of acute Q fever was established. Moreover, the serological test of indirect immunofluorescence assay (IFA) of the *C. burnetii* antibody was further performed in the Centers for Disease Control, certifying the result of mNGS. The patient was ultimately treated with doxycycline and recovered well. mNGS is an unbiased and comprehensive method in infrequent or culture-negative pathogen identification. To our knowledge, this is the first case of acute Q fever identified by mNGS and confirmed by IFA in Taizhou, China. A further large-scale prospective clinical cohort study is worth carrying out to compare the diagnostic efficiency of mNGS with traditional serological methods and PCR in acute Q fever.

## Introduction

Q fever is a globally distributed zoonotic infectious disease caused by *Coxiella burnetii*, first reported during a 1935 outbreak in Queensland, Australia (1). *Coxiella burnetii*, an obligate intracellular eosinophilic Gram-negative coccobacillus, belongs to the Legionellales order and Coxiellaceae family (2). There are two different antigenic forms of *C. burnetii*, divided into phase I and phase II according to the composition of membrane lipopolysaccharide (LPS) (3). Phase I *C. burnetii* is a significant virulence factor containing complete antigenic components and LPS. Phase II was formed after repeated passage through cell culture or chicken embryos, containing crude LPS and reduced virulence (4). Antiphase II antibodies can be detected 7 to 15 days after the onset of *C. burnetii* infection and then decrease within 3 to 6 months. It is generally believed that the titer of antiphase II IgG ≥220 and/or IgM ≥50 is of great significance for diagnosing primary Q fever infection. While the elevated IgG titers of ≥1:800 of antiphase I antibodies are related to chronic Q fever (5).

*C. burnetii* is characterized by solid virulence, aerosol transmission, easy storage, intense physical and chemical factors resistance, and long-term survival in the external environment (6). As a result, *C. burnetii* can widely infect humans and multiple animals through aerosols, so it has been identified as one of the crucial biological warfare agents and bioterrorism agents by relevant international organizations (7). In humans, the typical clinical symptoms of acute Q fever are influenza-like manifestations, including hyperpyrexia, chills, and severe headache (8, 9). Critical patients often present with pneumonia, granulomatous hepatitis, heart damage, neurological symptoms, and so on (10). If the treatment is not timely, about 5% of acute patients could turn to chronic Q fever, which can be manifested as endocarditis, chronic hepatitis, vascular infections, or osteomyelitis, increasing the difficulty of cure (11–13).

Early diagnosis and treatment will improve the prognosis of patients with Q fever. At present, the diagnosis of Q fever is mainly based on serological methods and polymerase chain reaction (PCR) (14, 15). However, the laboratory of many hospitals has not carried out the inspection items for Q fever. Furthermore, the clinical symptoms of acute Q fever are not typical, leading to a high rate of misdiagnosis. In recent years, the sequencing technology is shifting from basic research to clinical application benefited by the rapid development of science. Metagenomic next-generation sequencing (mNGS), with the strength of unbiased, broad coverage, and short time consuming, has become an attractive strategy in pathogens identification (16). Herein, we report a male patient with unknown fever, headache, and hepatic injury. *C. burnetii* was finally identified in this patient's peripheral blood by mNGS and confirmed through indirect immunofluorescence assay (IFA). The patient was treated with doxycycline, and he recovered well at follow-up.

## Case Presentation

A 48-year-old previously healthy man, who had a fever for 1 week, was admitted to our hospital's department of infectious diseases on February 25, 2021. Before arriving, he had been experiencing recurrent hyperpyrexia with a maximum temperature of 39.1°C, accompanied by chilly dizziness and headache. At the local hospital, laboratory tests showed an elevated C-reactive protein (CRP) level of 29.35 mg/L. Although receiving intravenous cephalosporin antibiotics for 3 days, his condition didn't improve.

**Figure 1:**
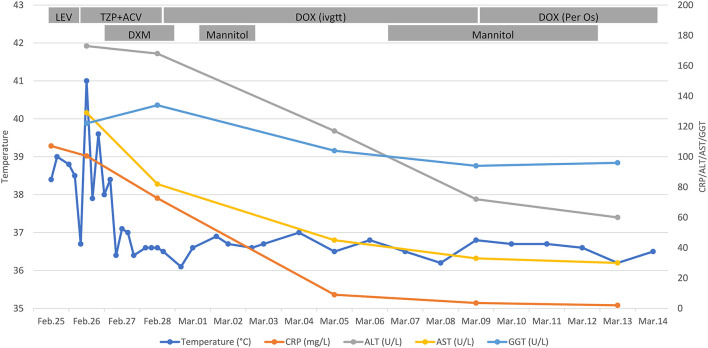
Clinical course of the 48-year-old patient. Timeline showed the dynamic monitoring of the patient's body temperature, and the level of CRP, ALT, AST, GGT. Besides, daily treatment schemes of the patient were recorded. CRP, C-reactive protein; ALT, alanine transaminase; AST, aspartate aminotransferase; GGT, gamma-glutamyltransferase; LEV, levofloxacin; TZP, piperacillin-tazobactam; ACV, acyclovir; DOX, doxycycline; DXM, dexamethasone; ivgtt, intravenously guttae.

After hospitalization, the patient presented hyperpyrexia, chill, nausea, dry cough, along with severe headache. Physical examination revealed tenderness under the xiphoid process and percussion pain in the hepatic region. Routine laboratory examinations were carried out promptly. It found that the level of CRP reached 107.8 mg/L (normal range 0–8 mg/L) (Figure 1). Besides, the patient presented with hepatic injury with elevated alanine transaminase (ALT) of 173U/L (normal range 9–60 U/L), aspartate aminotransferase (AST) of 129U/L (normal range 15–45 U/L), and gamma-glutamyltransferase (GGT) of 122 U/L (normal range 10–60 U/L). In contrast, the count of white blood cells (7.3 × 10^9^/L) and the percentage of neutrophils (63.5%) were in the normal range. Imaging examinations were performed subsequently. Chest computerized tomography (CT) scan showed localized emphysema and multiple small nodules in both lungs. And CT scan of the whole abdomen observed no apparent abnormality. Moreover, there was also no remarkable aberrant in the cranial magnetic resonance imaging and the cerebrovascular magnetic resonance angiography. The patient was empirically administrated with levofloxacin (0.5 g, qd) intravenously. And compound glycyrrhizin injection combined with reduced glutathione injection were used for conventional liver-protecting treatment. However, he still had repeated chill, hyperpyrexia, headache, and nausea. To further identify the pathogenic microorganism, peripheral blood sample from the patient was collected for mNGS (Supplementary Material).

On February 26, the patient presented with involuntary shaking of the right hand. Lumbar puncture was immediately performed, and his intracranial pressure was 150 mmH_2_O (normal range 80–180 mmH_2_O). Routine testing of cerebrospinal fluid (CSF) suggested a white blood cell count of 30/μL (normal range 0–8 /μL) and a red blood count of 10/μL (normal value 0 /μL). Besides, the bacterial culture of CSF was negative. Mannitol (100 mL) was applied to reduce the intracranial pressure. Despite adjusting the anti-infection regimen to intravenous piperacillin-tazobactam (4.5 g, q8h), his temperature remained poorly controlled with a maximum of 40.1°C. Other examinations, including blood culture, mycobacterial culture, Widal test, viral hepatitis markers, antinuclear antibody, antineutrophil cytoplasmic antibody, and thyroid function, all suggested no abnormality. Given the patient's medical history, symptoms, signs, and auxiliary examinations, it was initially considered encephalitis. Next, the patient was treated with intravenous acyclovir (0.5 g, q8h) and dexamethasone (10 day1, 5 mg day2). Then, his headache was relieved, and his body temperature decreased.

**Figure 2:**
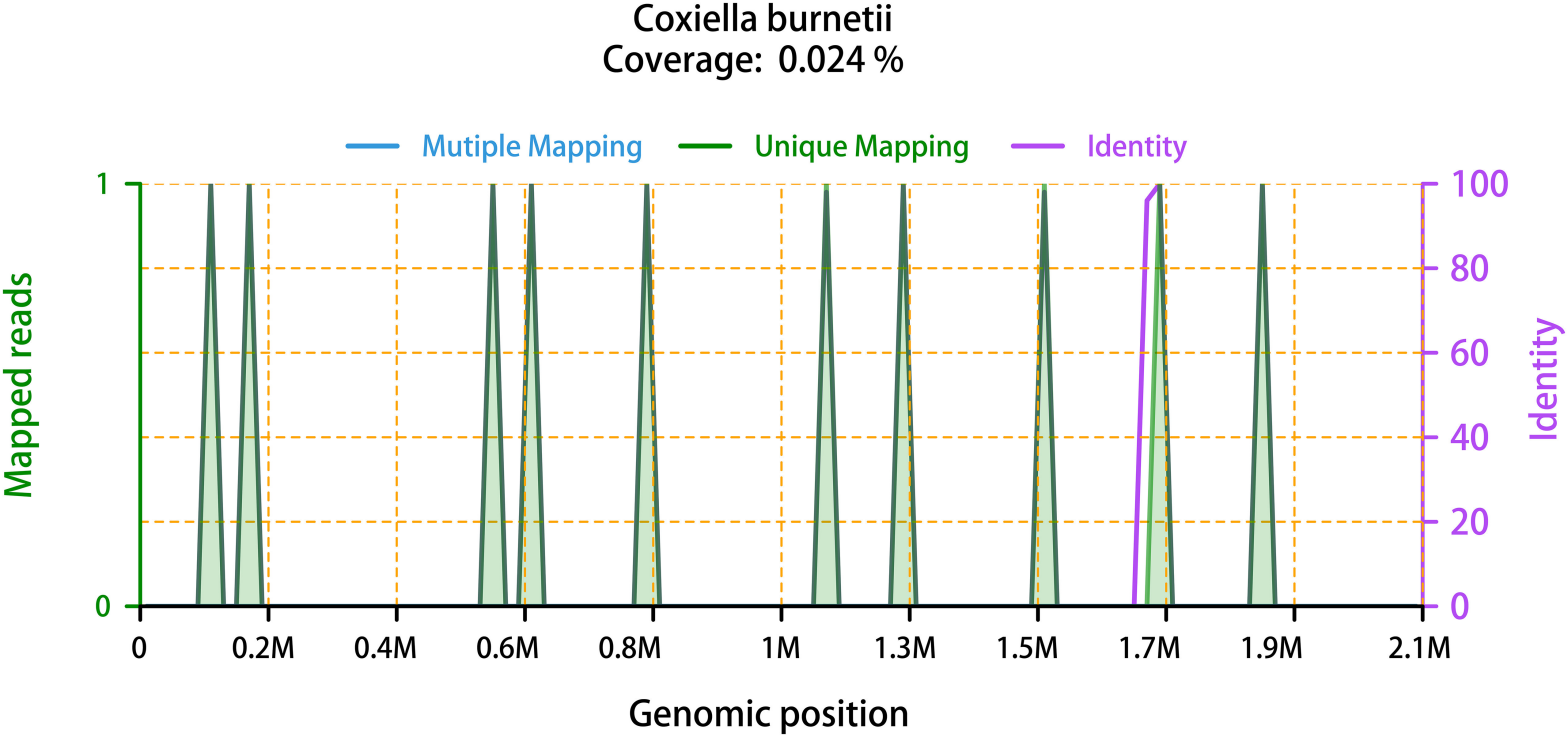
The mapped reads of *C. burnetii*, with the genome coverage of 0.024%. It showed the distribution of ten “Unique mapping” reads of *C. burnetii* identified by mNGS in the overall genome of *C. burnetii*. The ten reads covered 0.024% of *C. burnetii* genome. “Mutiple Mapping” indicates that the read can be mapped with the genome of *C. burnetii*, but not unique. “Unique Mapping” means that the read can be uniquely mapped to the genome of *C. burnetii*, but cannot be mapped to other species. Each read of “Unique Mapping” has its corresponding “Identity”. One purple line that represents “Identity” is at around 1.7 M genomic position. Another nine of the ten “Identity” curves are 100% overlapping with the green “Unique Mapping” curves, thus, they are not visible in this figure.

On February 28, it was reported that ten sequence reads of *Coxiella burnetii* were detected by mNGS through Illumina NextSeq 550 platform. The detail methodology related to mNGS was presented in Supplementary Material. The process of mNGS generated a total of 15,292,371 nucleic acid sequence reads. After filtering human host genomes, the amount of 65,757 microbial reads was obtained. The total number of bases in the genome of *C. burnetii* was 2,094,377 bp, and the total sequence length of *C. burnetii* measured in this study was 500 bp, covering 0.024% of the *C. burnetii* genome (Figure 2). Furthermore, mNGS was also performed on the patient's CSF sample to detect potential RNA viruses but none was found. Due to limitation of cost, DNA was not extracted from the CSF sample for mNGS. Asking about the epidemiological history, we learned that the patient drove to Zhaozhuang slaughterhouse in Linquan, Anhui province, to buy fresh beef half a month ago. After touching the beef, he did not wash his hands. We inferred that the patient was most likely infected by inhaling aerosols containing *C. burnetii* at the slaughterhouse. Combining with the clinic manifestations, the patient was diagnosed with acute Q fever. Instantly, the patient's serum sample was collected for antibody detection of *C. burnetii* by IFA in Zhejiang Provincial Center for Disease Control and Prevention, and the reported antibody titers of IgG were 1:256, confirming the result of mNGS. Piperacillin-tazobactam and acyclovir were discontinued, and doxycycline (0.1 g, q12h) was immediately used intravenously for anti-infective therapy for ten days. Orally doxycycline capsules (0.1 g, bid) were subsequently administrated for 6 days. After treatment, the patient didn't have a fever again. Laboratory tests conducted on March 13 indicated that the level of CRP, ALT, AST have returned to the normal range. Before being discharged, the blood sample was again collected for antibody detection of *C. burnetiid* through IFA. The result showed antibody titers of IgG was 1:1,024, which further verified the diagnosis of acute Q fever. The patient was followed up in the outpatient department 1 month later, and he recovered well.

## Discussion

*C. burnetii* is a pathogenic bacterium that can cause Q fever in humans. The host of *C. burnetii* ranges widely, including humans and domestic animals. Domestic ruminants are considered the primary source of infection of human Q fever, which can excrete pathogens through reproductive secretions, urine, feces, and milk (17). *C. burnetii* can also be transmitted through aerosols to practitioners in slaughterhouses and meat processing plants. In addition, a variety of ticks can carry *C. burnetii*, which play a role in the transmission between animals (18, 19). The patient had an epidemiological history of visiting a slaughterhouse to purchase fresh beef in our case. We inferred that the patient was likely infected by inhaling *C. burnetii* aerosols at the slaughterhouse. Q fever is still a severely neglected infectious disease in the global animal disease detection system, and Q fever's daily monitoring and diagnosis are insufficient (20–23). Therefore, the effective prevention of zoonotic diseases is significant to public health safety (24). Necessary measures need to be taken to control high-risk staff and livestock susceptible to Q fever.

*C. burnetii* doesn't grow under the circumstance of normative routine laboratory culture techniques and can only be handled in biosafety level III laboratories (25). Hence, the diagnosis of *C. burnetii* mainly relies on indirect diagnostic tools. A previous study compared the commercially available enzyme-linked immunosorbent test (ELISA), indirect fluorescent antibody test (IFAT), and complement fixation test (CFT) using the acute and convalescent serum of 126 patients with acute Q fever confirmed by PCR (26). It was found that the three serological tests were equally effective in diagnosing acute Q fever within 3 months of symptom onset. In follow-up visits, IFAT detected more IgG antibodies in serum than ELISA or CFT, making IFAT more suitable for pre-vaccination screening procedures. For chronic Q fever, Brendan and his colleagues have compared the serology and PCR results of three international reference laboratories on a clearly defined cohort of patients with Q fever (27). The results showed that the consistency of interpretation of the micro-immunofluorescence results of the three centers was only 35%, and it is recommended to develop an international standard for Q fever serological investigation.

In this case, mNGS rapidly identify the pathogenic *C. burnetii*, which provided significant assistance in early diagnosis for acute Q fever. As an emerging technology, mNGS has the advantages of no assumptions, wide-coverage, fast, and convenience (28). mNGS has been increasingly applied in identifying pathogenic microorganisms that are rare or difficult to cultivate clinically. Recent research reported an epidemic of Q fever confirmed by mNGS in 2018–2019 in Zhuhai city (29). Besides, Maiko's study conducted next-generation sequencing of plasma microbial cell-free DNA on a patient with prosthetic pulmonary valve culture-negative endocarditis to quickly diagnosis and genotyping of *C. burnetii*, to ensure early targeted therapy before valve replacement surgery (30). Furthermore, Huang et al. (31) has reported a case of tick-transmitted Q fever diagnosed by next-generation sequencing in Lishui, China, which lacks serological verification. Latest published research described a case of *Coxiella burnetii*-caused wound infection, which was determined via mNGS from the wound tissue, and validated through Sanger sequencing and serologic test (32). In our study, mNGS detected the *Coxiella burnetii* from the patient's peripheral blood, and the result of mNGS was subsequently confirmed by the traditional serological method of indirect immunofluorescence assay, verifying the reliability of mNGS. In spite of this, a relative high cost, limited accessibility, and finite real-world positive clinical impact associated with mNGS could limit its routine application in clinical practice (33). Further development of the mNGS technology and its establishment of clinical standardized application procedure will promote the universal popularization of mNGS in detecting pathogenic microorganisms (34).

The patient in our case was a previous healthy middle-aged male and treated with doxycycline. It's investigated that doxycycline treatment positively correlates with faster fever reduction (35). Another research found it may prevent Q fever from progressing to persistent focalized infection when doxycycline was administered at the time of primary infection (36). Furthermore, a recent retrospective study observed that delays more than 7 days in starting treatment would have a higher hospitalization rate (37). Therefore, early diagnosis and treatment of Q fever are of great significance. Once the patient is identified as having potential risk factors for endocarditis, vascular infections, and immune deficiency, individualized treatment, and follow-up strategies are needed to control disease progression (17).

## Limitations

There are some limitations of this case. First, we did not collect environmental and animal samples from the Zhaozhuang slaughterhouse in Linquan, Anhui, for mNGS. As a result, we cannot confirm the origin of the bacterial exposure. Second, mNGS detected only ten reads covering 0.024% of the *C. burnetii* genome in this study. This could be due to a low pathogen load in the sample, an insufficient amount of the total nucleic acid extracted, or a high amount of human's genomic material relative to pathogens. In the future clinical application, the processes of specimen collection, nucleic acid extraction, sequencing, and bioinformatics analysis should be more standardized, to improve the reliability of results. Third, a large-scale prospective clinical cohort study should be carried out to compare the sensitivity and specificity of mNGS with traditional serological methods and PCR in the diagnosis of acute Q fever.

## Conclusion

In summary, we report the first case of acute Q fever identified by mNGS and confirmed by indirect immunofluorescence assay in Taizhou, China. mNGS has become an attractive pathogen identification strategy and can provide an efficient early diagnosis of acute Q fever. With the development of high-quality clinical studies on mNGS and the formulation of global specialist consensuses on the clinical application of mNGS, mNGS is expected to further assist clinical diagnosis and treatment in the future.

## Data Availability Statement

The datasets presented in this study can be found in online repositories. The names of the repository/repositories and accession number(s) can be found below: https://db.cngb.org/, CNP0002548.

## Ethics Statement

The studies involving human participants were reviewed and approved by the Clinical Ethics Committee of Taizhou Municipal Hospital (2021-LW124). The patients/participants provided their written informed consent to participate in this study. Written informed consent was obtained from the individual(s) for the publication of any potentially identifiable images or data included in this article.

## Author Contributions

BZ and YY designed the study. YY collected the data and performed the data analyses. QJ was involved in patient management and provided clinical information. ZY performed the laboratory test of indirect immunofluorescence assay. JH conceptualized this work. WL performed bioinformatic analysis. QS wrote the original manuscript. JM provided medication guidance and revised the manuscript. BZ revised and supervised the paper. All authors contributed to the article and approved the final manuscript.

## Funding

This study was partially funded by grants from the National Natural Science Foundation of China (No. 82072314) and the Research Project of Jinan Microecological Biomedicine Shandong Laboratory (JNL-2022011B).

## Conflict of Interest

WL was employed by Realbio Genomics Institute. JH was employed by Sansure Biotech Inc. The remaining authors declare that the research was conducted in the absence of any commercial or financial relationships that could be construed as a potential conflict of interest.

## Publisher's Note

All claims expressed in this article are solely those of the authors and do not necessarily represent those of their affiliated organizations, or those of the publisher, the editors and the reviewers. Any product that may be evaluated in this article, or claim that may be made by its manufacturer, is not guaranteed or endorsed by the publisher.

## References

[1] Derrick E. “Q” fever, a new fever entity: clinical features, diagnosis and laboratory investigation. Rev Infect Dis. (1983) 5:790–800. 10.1093/clinids/5.4.790 6622891

[2] Fournier P Marrie T Raoult D. Diagnosis of Q fever. J Clin Microbiol. (1998) 36:1823–34. 10.1128/JCM.36.7.1823-1834.1998 9650920 PMC104936

[3] Maurin M Raoult D. Q fever. Clin Microbiol Rev. (1999) 12:518–53. 10.1128/CMR.12.4.518 10515901 PMC88923

[4] Abnave P Muracciole X Ghigo E. Coxiella burnetii lipopolysaccharide: what do we know? Int J Mol Sci. (2017) 18:2509. 10.3390/ijms18122509 29168790 PMC5751112

[5] Parker N Barralet J Bell A. Q fever. Lancet. (2006) 367:679–88. 10.1016/S0140-6736(06)68266-4 16503466

[6] Long C Beare P Cockrell D Larson C Heinzen R. Comparative virulence of diverse Coxiella burnetii strains. Virulence. (2019) 10:133–50. 10.1080/21505594.2019.1575715 30782062 PMC6389282

[7] Madariaga M Rezai K Trenholme G Weinstein R. Q fever: a biological weapon in your backyard. Lancet Infect Dis. (2003) 3:709–21. 10.1016/S1473-3099(03)00804-1 14592601

[8] Heo J Choi Y Kim E Lee S Lim S Hwang S. Clinical characteristics of acute Q fever patients in South Korea and time from symptom onset to serologic diagnosis. BMC Infect Dis. (2019) 19:903. 10.1186/s12879-019-4479-0 31660875 PMC6819606

[9] Finn T Babushkin F Geller K Alexander H Paikin S Lellouche J. Epidemiological, clinical and laboratory features of acute Q fever in a cohort of hospitalized patients in a regional hospital, Israel, 2012-2018. PLoS Negl Trop Dis. (2021) 15:e0009573. 10.1371/journal.pntd.0009573 34264953 PMC8315502

[10] Budgin A Abidi M Bajrovic V Miller M Johnson S. Severe acute Q fever pneumonia complicated by presumed persistent localized Q fever endocarditis in a renal transplant recipient: A case report and review of the literature. Transplant Infect Dis. (2020) 22:e13230. 10.1111/tid.13230 31808240

[11] Kouijzer I Oyen W Bleeker-Rovers C. Vascular infection with outside-in vertebral destruction in chronic Q fever. Lancet Infect Dis. (2018) 18:603. 10.1016/S1473-3099(18)30272-X 29856356

[12] van Roeden S Wever P Kampschreur L Gruteke P van der Hoek W Hoepelman A. Chronic Q fever-related complications and mortality: data from a nationwide cohort. Clin Microbiol Infect. (2019) 25:1390–8. 10.1016/j.cmi.2018.11.023 30543852

[13] Buijs S Bleeker-Rovers C van Roeden S Kampschreur L Hoepelman A Wever P. Still new chronic Q fever cases diagnosed eight years after a large Q fever outbreak. Clin Infect Dis. (2021) 73:1476–83. 10.1093/cid/ciab476 34028546

[14] Schneeberger P Hermans M van Hannen E Schellekens J Leenders A Wever P. Real-time PCR with serum samples is indispensable for early diagnosis of acute Q fever. Clin Vaccine Immunol. (2010) 17:286–90. 10.1128/CVI.00454-09 20032219 PMC2815520

[15] Wielders C van Loenhout J Morroy G Rietveld A Notermans D Wever P. Long-term serological follow-up of acute Q-Fever patients after a large epidemic. PLoS ONE. (2015) 10:e0131848. 10.1371/journal.pone.0131848 26161658 PMC4498618

[16] Dulanto Chiang A Dekker JP. From the pipeline to the bedside: advances and challenges in clinical metagenomics. J Infect Dis. (2020) 221:S331–40. 10.1093/infdis/jiz151 31538184 PMC7325616

[17] Eldin C Mélenotte C Mediannikov O Ghigo E Million M Edouard S. From Q fever to coxiella burnetii infection: a paradigm change. Clin Microbiol Rev. (2017) 30:115–90. 10.1128/CMR.00045-16 27856520 PMC5217791

[18] Ni J Lin H Xu X Ren Q Aizezi M Luo J. Coxiella burnetii is widespread in ticks (Ixodidae) in the Xinjiang areas of China. BMC Vet Res. (2020) 16:317. 10.1186/s12917-020-02538-6 32859190 PMC7455992

[19] Jiao J Zhang J He P OuYang X Yu Y Wen B. Coxiella burnetiiIdentification of tick-borne pathogens and genotyping of in in Yunnan Province, China. Front Microbiol. (2021) 12:736484. 10.3389/fmicb.2021.736484 34621258 PMC8491607

[20] Delsing C Kullberg B Bleeker-Rovers C. Q fever in the Netherlands from 2007 to 2010. Neth J Med. (2010) 68:382–7. 21209463

[21] El-Mahallawy H Lu G Kelly P Xu D Li Y Fan W. Q fever in China: a systematic review, 1989-2013. Epidemiol Infect. (2015) 143:673–81. 10.1017/S0950268814002593 25274488 PMC9507106

[22] Gidding H Faddy H Durrheim D Graves S Nguyen C Hutchinson P. Seroprevalence of Q fever among metropolitan and non-metropolitan blood donors in New South Wales and Queensland, 2014-2015. Med J Aust. (2019) 210:309–15. 10.5694/mja2.13004 30848517

[23] Kim Y Jeong H Kim D Huh K Choi S Lee H. Epidemiological investigation and physician awareness regarding the diagnosis and management of Q fever in South Korea, 2011 to 2017. PLoS Negl Trop Dis. (2021) 15:e0009467. 10.1371/journal.pntd.0009467 34077423 PMC8202952

[24] Francis J Robson J. Q fever: more common than we think, and what this means for prevention. Med J Aust. (2019) 210:305–6. 10.5694/mja2.50024 30773646

[25] Anderson A Bijlmer H Fournier P Graves S Hartzell J Kersh G. Diagnosis and management of Q fever–United States, 2013: recommendations from CDC and the Q Fever working group. MMWR Recomm Rep. (2013) 62:1–30. 23535757

[26] Wegdam-Blans M Wielders C Meekelenkamp J Korbeeck J Herremans T Tjhie H. Evaluation of commonly used serological tests for detection of coxiella burnetii antibodies in well-defined acute and follow-up sera. Clin Vaccine Immunol. (2012) 19:1110–5. 10.1128/CVI.05581-11 22623653 PMC3393374

[27] Healy B van Woerden H Raoult D Graves S Pitman J Lloyd G. Chronic Q fever: different serological results in three countries–results of a follow-up study 6 years after a point source outbreak. Clin Infect Dis. (2011) 52:1013–9. 10.1093/cid/cir132 21460316

[28] Li N Cai Q Miao Q Song Z Fang Y Hu B. High-throughput metagenomics for identification of pathogens in the clinical settings. Small Methods. (2021) 5:2000792. 10.1002/smtd.202000792 33614906 PMC7883231

[29] Huang M Ma J Jiao J Li C Chen L Zhu Z. The epidemic of Q fever in 2018 to 2019 in Zhuhai city of China determined by metagenomic next-generation sequencing. PLoS Negl Trop Dis. (2021) 15:e0009520. 10.1371/journal.pntd.0009520 34264939 PMC8282036

[30] Kondo M Dalai S Venkatasubrahmanyam S Eisenberg N Robinson B Westblade L. Coxiella burnetiidiagnosis and genotyping of endocarditis in a patient with prosthetic pulmonary valve replacement using next-generation sequencing of plasma microbial cell-Free DNA. Open Forum Infect Dis. (2019) 6:ofz242. 10.1093/ofid/ofz242 31249846 PMC6580995

[31] Huang J Wang R Gao C Lü Y Cao Z Deng S. A case of tick-transmitted Q fever in Lishui, China diagnosed by next-generation sequencing. J Int Med Res. (2021) 49:3000605211025398. 10.1177/03000605211025398 34590876 PMC8489766

[32] Zhang J Hao Y Wang Z Yang Q. Diagnosis of coxiella burnetii infection via metagenomic next-generation sequencing: a case report. BMC Infect Dis. (2022) 22:373. 10.1186/s12879-022-07309-2 35418079 PMC9008969

[33] Hogan CA Yang S Garner OB Green DA Gomez CA Dien Bard J. Clinical impact of metagenomic next-generation sequencing of plasma cell-free DNA for the diagnosis of infectious diseases: a multicenter retrospective cohort study. Clin Infect Dis. (2021) 72:239–45. 10.1093/cid/ciaa035 31942944

[34] Chiu CY Miller SA. Clinical metagenomics. Nat Rev Genet. (2019) 20:341–55. 10.1038/s41576-019-0113-7 30918369 PMC6858796

[35] Sobradillo V Zalacain R Capelastegui A Uresandi F Corral J. Antibiotic treatment in pneumonia due to Q fever. Thorax. (1992) 47:276–8. 10.1136/thx.47.4.276 1585291 PMC463691

[36] Kampschreur L Dekker S Hagenaars J Lestrade P Renders N de Jager-Leclercq M. Identification of risk factors for chronic Q fever, the Netherlands. Emerg Infect Dis. (2012) 18:563–70. 10.3201/eid1804.111478 22469535 PMC3309671

[37] Dijkstra F Riphagen-Dalhuisen J Wijers N Hak E Van der Sande M Morroy G. Antibiotic therapy for acute Q fever in The Netherlands in 2007 and 2008 and its relation to hospitalization. Epidemiol Infect. (2011) 139:1332–41. 10.1017/S0950268810002621 21087542

