# Supplementary material for: Case Report: Metagenomic Next-Generation Sequencing Clinches the Diagnosis of Acute Q Fever and Verified by Indirect Immunofluorescence Assay
Source: Front Med (Lausanne). 2022 May 26;9:846526. doi: 10.3389/fmed.2022.846526 (PMC9204269; doi:10.3389/fmed.2022.846526)
Supplement: Supplementary file 1 [file Presentation_1.PDF]

MATERIALS AND METHODS

Patient and Sample

The patient involved in our study was a 48-year-old man with recurrent unknown fever for a week. We collected related information of the patient's demographic characteristics, symptom, sign, laboratory test and imaging examination results, diagnosis and treatment course, and follow-up state. Peripheral blood sample and cerebrospinal fluid (CSF) sample from the patient was collected on the basis of standard operating procedure. This study was reviewed and approved by the Clinical Ethics Committee of Taizhou Municipal Hospital (2021-LW124).

Metagenomic Next-generation Sequencing Protocol

First, DNA extraction from blood sample was performed by TIANamp Micro DNA kit. QIAamp Virus RNA kit was used for RNA extraction from CSF sample. The nucleic acid was quantified by Qubit™ Flex Fluorometer (Thermo Fisher Scientific, USA), and the purity of nucleic acid was measured by NanoDrop Spectrophotometer (Thermo Fisher Scientific, USA). Second, we performed library construction and the library fragment size is quality-controlled using Agilent 2100 bioanalyzer (Agilent, USA). Third, the qualified library was sequenced on NextSeq550 platform (Illumina, USA) with a single-end 75bp sequencing strategy. Forth, quality control, removal of low-quality reads, and human host genome subtraction of the raw sequencing data was conducted. Finally, we performed bioinformatic analysis based on the microbial reference database RealSeq-PM. After the sequence alignment, we obtained the absolute sequence reads and relative abundance for each identified microbe.
